# Deciphering the Role of Sirtuin‐1 Gene Polymorphism in Diabetic Nephropathy: A Systematic Review and Meta‐Analysis

**DOI:** 10.1155/jdr/5528647

**Published:** 2026-01-29

**Authors:** Hira Moin, Munazza Asad, Maaz Waseem, Sarim Zafar, Hania Syed, Ramsha Syed, Momina Hussain

**Affiliations:** ^1^ Department of Physiology, NUST School of Health Sciences, National University of Sciences and Technology (NUST), H-12 Sector, Islamabad, Pakistan, nust.edu.pk; ^2^ Department of Microbiology and Biotechnology, Atta-ur-Rehman School of Applied Biosciences, National University of Sciences and Technology (NUST), H-12 Sector, Islamabad, Pakistan, nust.edu.pk; ^3^ Department of Medicine, Al-Nafees Medical College, Isra University, Islamabad, Pakistan, isra.edu.pk; ^4^ Department of Surgery, Fauji Foundation Hospital, Rawalpindi, Pakistan; ^5^ Chinese Academy of Tropical Agricultural Sciences, Sanya, China, catas.cn

**Keywords:** diabetic nephropathy, genetic susceptibility, meta-analysis, polymorphism, renal disease, risk stratification, sirtuin-1

## Abstract

Sirtuin‐1‐gene (*SIRT1*) plays a key role in regulating metabolic and inflammatory processes. This review is aimed at evaluating the association between *SIRT1*‐polymorphisms and diabetic nephropathy susceptibility. Observational‐cohort and case‐control studies were included. Data extraction followed PRISMA 2020 guidelines, and study quality was assessed using the Newcastle‐Ottawa Scale. Meta‐analysis was done using a random‐effects model, with heterogeneity and publication bias assessed using *I* ^2^ statistics and funnel plots. Subgroup analyses were done by ethnicity and genotyping methods. Meta‐analysis showed *SIRT1*‐polymorphisms rs7895833 (OR: 2.71, 95% CI: 2.67–2.76) and rs2273773 (OR: 1.51, 95% CI: 1.16–1.97) to be significantly associated with increased DN risk. rs7069102 was not significantly associated (OR: 1.12, 95% CI: 0.80–1.59). Subgroup analysis showed population‐specific variations with stronger associations in Chinese and Indian populations. Sensitivity analysis maintained results′ robustness, though funnel plot analysis suggested potential publication bias. Conclusively, *SIRT1* polymorphisms, particularly rs7895833 and rs2273773, are associated with DN susceptibility, confirming their potential as genetic markers for DN risk stratification.

## 1. Introduction

Diabetic nephropathy (DN) is a severe complication of diabetes mellitus and remains one of the major causes of end‐stage renal disease (ESRD) globally [1]. DN is characterized by progressive kidney injury and is clinically manifested by increased albuminuria, reduced glomerular filtration rate (eGFR), and ultimately renal failure if left untreated [2]. The prevalence of DN is rising in parallel with the diabetes epidemic globally and has enormous healthcare, economic, and societal burdens. Despite clinical improvement, DN continues to be an adverse prognostic factor on patient outcomes, underscoring the necessity for an immediate clarification of its multifactorial pathogenesis and discovery of solid markers for early risk stratification and treatment [3, 4].

Genetic susceptibility is a key regulator of the susceptibility and development of DN, both on diabetic kidney disease formation and severity [5]. In the last decades, large research studies have identified many candidate genes implicated in DN pathophysiology and reported significant genetic heterogeneity of disease expression. Among those candidate genes, sirtuin‐1 (*SIRT1*) has drawn widespread interest due to its participation in multifunctional biological activities related to cellular aging, metabolism, inflammation, and oxidative stress processes all critical in DN progression. SIRT1, a Class III histone deacetylase, plays roles in protective renal homeostatic mechanisms through modulating apoptosis, fibrosis, inflammation, and metabolic regulation of renal tissue [6, 7].

The preclinical and clinical findings emphasize the potential protective effects of SIRT1 against renal injury. Animal models demonstrate that activation or overexpression of SIRT1 confers renal protection through the reduction of inflammation, oxidative stress, and fibrosis, which are crucial pathophysiologic processes of DN development [8]. In diabetic animal models, pharmacological or genetic activation of SIRT1 significantly reduces albuminuria, glomerular injury, and tubular damage. Translational studies also show that reduced renal *SIRT1* expression is strongly correlated with DN severity in diabetic patients. Collectively, these findings suggest that SIRT1 is a potential therapeutic target and biomarker for DN prevention and treatment [8, 9].

Polymorphisms of the *SIRT1* gene have been extensively investigated for their potential implications in susceptibility and progression of DN. *SIRT1* gene SNPs such as rs7895833, rs2273773, and rs7069102 were directly examined among different populations for association with DN risk [10, 11]. However, findings within individual research studies have proven to be inconsistent, most likely because of variability in genetic backgrounds, sample sizes, study designs, and methodological heterogeneity. Furthermore, genetic association studies of DN also often present reduced statistical power when taken in isolation, especially in populations characterized by heterogeneous genetic architectures. Therefore, the exclusive effect of *SIRT1* polymorphisms on DN susceptibility and progression has not yet been determined, necessitating critical synthesis through systematic processes [12].

In order to explain such discrepancies and provide a firm estimate of the association between *SIRT1* polymorphisms and DN, performing a systematic review and meta‐analysis is imperative. Meta‐analytical techniques facilitate statistical aggregation between studies, with greater power to detect significant relationships and reduce random error within individual studies. Meta‐analyses also facilitate investigation of the source of possible heterogeneity as well as subgroup analyses to investigate effects stratified by such variables as ethnicity, diabetes type, and genotyping strategy, thus providing precise details of population‐specific risk profiles [13].

Considering the notable differences seen between ethnic groups in genetic susceptibility to DN, it is important to investigate possible population‐specific variations in the relationship between *SIRT1* polymorphisms and risk of DN [13]. Earlier research has indicated that genetic susceptibility to DN may differ considerably between populations, possibly affecting disease screening practices, therapeutic approaches, and personalized medicine strategies. Knowledge of whether particular *SIRT1* polymorphisms have more or less association in specific ethnic groups will allow more precise risk stratification and interventions [14].

Furthermore, elucidating how *SIRT1* polymorphisms affect disease progression biomarkers such as eGFR decline and albuminuria will explain the pathogenesis of DN. Albuminuria and decreased eGFR are critical clinical markers used for DN diagnosis, monitoring, and prediction [14]. Investigating the genetic effect of *SIRT1* variants on these clinical outcomes will provide insight into how genetic heterogeneity influences renal disease outcome in diabetic patients, thereby informing precision medicine interventions aimed at preventing DN progression.

Therefore, this meta‐analysis and review is aimed at integrating existing genetic association studies to examine comprehensively the association between *SIRT1* gene polymorphisms and susceptibility to DN. Some of the goals are to compute the pooled effect size (odds ratio [OR] and confidence intervals [CIs]), conduct subgroup analysis by ethnicity, diabetes type, and study type, evaluate *SIRT1* polymorphism effects on markers of DN progression, and systematically identify heterogeneity and publication bias. By clarifying the role of *SIRT1* gene variants in DN, this study will significantly contribute to the development of personalized medicine regimens, enabling early identification of at‐risk individuals and directing therapy protocols aimed at preventing or slowing DN development and progression.

## 2. Methods

### 2.1. Study Design and Protocol Registration

The research is a meta‐analysis and systematic review with its design aligned in line with Preferred Reporting Items for Systematic Reviews and Meta‐Analyses guidelines. The protocol of review was prospectively registered on the International Prospective Register of Systematic Reviews (PROSPERO) for the aim of achieving methodological transparency along with avoiding any form of bias (Registration: CRD420251033898).

### 2.2. Eligibility Criteria (Inclusion and Exclusion Criteria)

Those studies which satisfied clearly defined criteria were selected. Observational studies (case‐control and cohort studies) comparing associations of *SIRT1* gene polymorphisms with DN in human diabetic patients with Type 1 or Type 2 diabetes were included based on inclusion criteria. Studies had to provide sufficient genetic and statistical data to compute effect sizes, for example, ORs, relative risks (RRs), or hazard ratios (HRs), with 95% CIs. Animal model studies without corresponding genetic polymorphism data, review articles, letters, editorials, conference abstracts with no full‐text available, and studies without sufficient quantitative outcome data that are required to be meta‐analyzed were excluded.

### 2.3. Information Sources and Literature Search Strategy

A careful and comprehensive literature search was conducted to identify relevant studies assessing the association of *SIRT1* gene polymorphisms with DN. Electronic databases like PubMed, Scopus, Web of Science, Embase, and Cochrane Library were extensively searched from their starting date until today. The search strategy employed specific terms and Medical Subject Headings (MeSH) terms to include “SIRT1,” “Sirtuin‐1,” “polymorphism,” “single nucleotide polymorphisms (SNPs),” “rs7895833,” “rs2273773,” “rs7069102,” “diabetic nephropathy,” “DN,” “kidney disease,” “renal impairment,” “albuminuria,” and “glomerular filtration rate (eGFR)”. Boolean operators such as “AND” and “OR” were utilized strategically to ensure maximum sensitivity and specificity of the searches. The literature search was restricted to peer‐reviewed, English language publications without restrictions in terms of publication date. Each search strategy used on respective database is presented in Table S1.

### 2.4. Study Selection Process

Two independent reviewers (HM and MA) screened all studies identified initially based on title and abstract. After that the full‐text detailed review for potentially eligible studies was performed (HM and MA) in compliance with the PRISMA 2020 guidelines [15]. Disagreement at any step was resolved by consensus meetings or by referring to a third reviewer (MW) if necessary.

### 2.5. Data Collection Process Including Data Items and Effect Measures

Data extraction was performed systematically and independently, extracting major information such as author names, year of publication, geographic location, study design, sample size, patient demographics, type of diabetes (Type 1 or Type 2 diabetes), genetic polymorphisms investigated (particularly rs7895833, rs2273773, and rs7069102), genotyping techniques, and clinical outcomes assessed (such as DN diagnosis, presence of albuminuria, and reduction in eGFR). Moreover, relevant statistical estimates, including ORs, RRs, HRs, CIs, and *p* values, were diligently downloaded to facilitate further quantitative examination. Additionally, non‐*SIRT1* polymorphism was included because DN is a complex disease and includes a number of pathways, which are inflammation, fibrosis, and oxidative stress. The presence of the other polymorphisms, including *TNF-a, IL-1b, eNOS, PARP-1, FOXO1*, and *IL-10* polymorphisms is reasonable since they play a role in the pathophysiology of DN [5, 16]. Many of these inflammatory and metabolic pathways interact with the *SIRT1* gene (that has a protective role in DN). As an example: TNF‐a and IL‐1b are inflammatory cytokines that are known to cause kidney damage in diabetes [16]. The eNOS (endothelial nitric oxide synthase) plays a vital role in the maintenance of vascular health [2, 5], in which the capacity directly influences the functioning of the kidneys and thus has connection to DN. PARP‐1 is involved in cell death and DNA repair, which is useful in diabetic kidney disease [2, 5]. FOXO1 and IL‐10 play a metabolic role and immune modulation, both of which have been found to play a role in DN progression [5, 16]. As DN is not a one‐to‐one interaction with *SIRT1* variants but it is a complicated interplay of genes, thus, their incorporation enables more thorough examination on genetic aspects as contributory to DN risk.

### 2.6. Risk of Bias and Quality Assessment

Methodological quality of included studies was comprehensively assessed using the Newcastle‐Ottawa Scale (NOS) that approximates study quality based on three categories: selection of study groups, comparability of groups, and ascertainment of outcome. Studies were then categorized into groups corresponding to low, moderate, or high quality according to NOS scoring guidelines. Publication bias was also systematically assessed using visual inspection of funnel plots and statistically using Egger′s regression test and Begg′s rank correlation test.

### 2.7. Statistical Analysis

Meta‐analytical techniques were employed to estimate pooled effect sizes for the interaction between *SIRT1* gene polymorphisms (rs7895833 [dominant model], rs2273773 [allele contrast], rs7069102 [codominant: recessive]) and risk of DN. ORs with related 95% CIs were estimated to quantify the size of genetic associations. Study heterogeneity was estimated by Cochran′s Q statistic and quantified by the *I* ^2^ statistic, which had scores greater than 50% indicating significant heterogeneity. A random‐effects model was utilized to account for heterogeneity across studies. Subgroup analyses were performed based on prespecified factors such as ethnicity, diabetes type (Type 1 diabetes vs. Type 2 diabetes), and genotyping strategies. Sensitivity analyses were performed with leave‐one‐out to evaluate the robustness of findings and to investigate the influence of individual studies on overall findings. All statistical testing was performed with comprehensive statistical meta‐analysis software, for example, Review Manager (RevMan) or Stata software. Statistical significance was defined at a *p* value of less than 0.05.

## 3. Results

### 3.1. Study Selection and Characteristics

**Figure 1:**
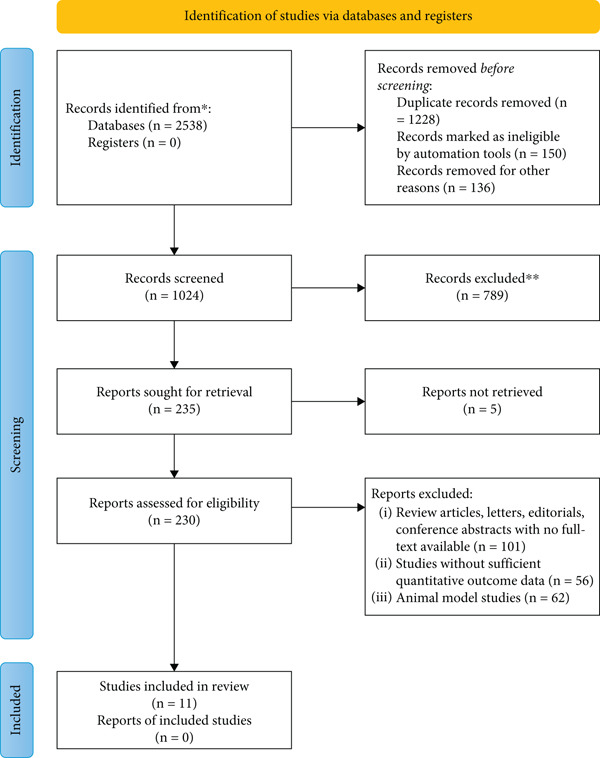
PRSIMA Flow diagram.

The systematic review and meta‐analysis of *SIRT1* gene polymorphism in DN explores the genetic difference in the *SIRT1* gene and susceptibility to DN, the most common complication of diabetes. The investigation does literature search and undertakes meta‐analysis using information from over one study to establish whether *SIRT1* polymorphisms play an essential role in the risk and development of DN. The PRISMA 2020 flow diagram shows that 11 studies were included after screening 2538 database records with 1514 records excluded due to duplication or ineligibility. Exclusions were primarily due to the lack of quantitative outcome data, review articles, or animal model‐based studies. The findings are valuable in explaining the genetic susceptibility of DN and may also facilitate the detection of potential genetic markers for risk stratification and therapeutic interventions among diabetic patients (Figure 1). The systematic review and meta‐analysis of *SIRT1* gene polymorphism in DN is aimed at exploring the association of some genetic forms of *SIRT1* with susceptibility to DN in Type 2 diabetes mellitus patients. The table provides an overview of key included studies, including sample size, study design type, population stratification, and genotyping methodologies. The study is based on different geographical sites in China, India, Slovenia, and Germany that encompass different ethnic populations. A number of *SIRT1* polymorphisms, such as rs10509291, rs12778366, rs4746720, and rs7069102, were evaluated using advanced genetic analysis techniques such as real‐time PCR, TaqMan assays, and PCR‐RFLP. The polymorphisms are reputed to modulate SIRT1 activity with potential impacts on the risk of DN by changing metabolic and inflammatory pathways associated with declining renal function. The findings suggest a possible ethnic and population‐specific heterogeneity of the association between *SIRT1* polymorphisms and DN susceptibility, as seen in Chinese Han, South Indian, and Caucasians. While variants such as rs4746720 and rs10823116 have been reported frequently in most studies, others such as rs7069102 and FOXO1 rs17446614 are more population‐specific. The mean age range among studies shows that most participants were middle‐aged to elderly, and both male and female subjects were involved, offering gender balance. These results highlight the need for additional large‐scale, multiethnic studies to confirm the role of *SIRT1* polymorphisms in DN, creating avenues for potential genetic screening, personalized therapeutic approaches, and precision medicine methods for diabetic patients at risk of nephropathy (Table 1).

**Table 1:** Summary of Studies Investigating *SIRT1* gene polymorphisms in diabetic nephropathy.

**Author and year**	**Country/region**	**Study design**	**Sample size**	**Diabetes type**	**Patient demographics**	**Genetic variants studied**	**Genotyping methods**	**Age**	**Gender**
[16]	India	Case‐control	448 DN cases, 414 T2DM without DN, 464 healthy controls	Type 2 diabetes	Indian population	TNF‐Î ± rs1800629, IL‐1Î^2^ rs16944, IL‐6 rs1800795	Sequence‐specific PCR	Mean~50‐60 years	mixed gender (male and female)
[17]	China	Case‐control	310 T2DM cases, 301 controls	Type 2 diabetes	Chinese Han population	SIRT1 rs10509291, rs12778366, rs10997870, rs10823112, rs4746720	Snapshot assay, DNA sequencing	> 40 years	Mixed gender (male and female)
[18]	Slovenia	Case‐control	301 DN cases, 423 controls (T2DM without DN)	Type 2 diabetes	Slovene (Caucasian) population	SIRT1 rs7069102	Real‐time PCR TaqMan	Adult (specific mean age not stated clearly)	Mixed gender (male and female)
[19]	India	Case‐control	155 DN cases, 162 T2DM controls without DN	Type 2 diabetes	South Indian population	ENOS 894G > T, PARP‐1 Val762Ala	PCR and TaqMan allele discrimination assay	Adult (specific mean age not stated clearly)	Mixed gender (male and female)
[20]	China	Case‐control	86 DN cases, 94 controls	Type 2 diabetes	Chinese population	TNF‐Î ± ‐308G/A	PCR‐SSP	Adult	Matched gender distribution
[21]	Germany	Case‐control	173 DN cases, 186 T2DM controls without DN	Type 2 diabetes	Caucasian (German population)	IL1b C‐511 T	PCR‐RFLP	Adult	Mixed gender (male and female)
[22]	China	Case‐control	Not specified clearly	Type 2 diabetes	Chinese Han population	SIRT1 rs4746720	TaqMan assay	Adult, > 65 years	Mixed gender (male and female)
[23]	China	Case‐control	172 DN cases, 344 controls	Type 2 diabetes	Chinese population	IL‐10 ‐592C/A, ‐819C/T, ‐1082A/G	PCR‐RFLP	Adult	Mixed gender (male and female)
[24]	China	Case‐control	150 DN cases, 160 T2DM controls	Type 2 diabetes	Chinese population	SIRT1 rs3818292, rs4746720, rs10823108	PCR‐RFLP	Mean~58 years	Mixed gender (male and female)
[25]	China	Case‐control	653 DN cases, 413 T2DM controls	Type 2 diabetes	Chinese Han population	SIRT1 rs3818292, rs4746720, rs10823108; FOXO1 rs17446614	PCR‐RFLP	Mean~62 years (cases), ~58 years (controls)	Mixed gender (male and female)
[26]	China	Case‐control	284 DN cases, 284 controls	Type 2 diabetes	Chinese elderly population	SIRT1 rs4746720, rs10509291, rs2236319, rs10823116	TaqMan assay	Elderly population	Mixed gender (male and female)

### 3.2. Clinical Outcomes and Genetic Associations

The figure illustrates the overall findings of various studies investigating the association between *SIRT1* gene polymorphism and DN in Type 2 diabetes mellitus patients. All studies investigated various gene polymorphisms, measuring the impact on clinical endpoints such as DN diagnosis, albuminuria, GFR, and proteinuria. The ORs or risk ratios with 95% CIs provide information on the potential genetic susceptibility to DN. The majority of these studies found statistically significant correlations (*p* values < 0.05), indicating that certain polymorphisms may be involved in DN susceptibility. For instance, SIRT1 rs10823112, rs4746720, and rs10509291 were involved in increased risk of DN, Han et al. [17] having an OR of 1.51 (1.152–1.994) for rs10823112 and an OR of 1.37 (1.037–1.819) for rs4746720, both of which were statistically significant.

Other studies also examined inflammatory and metabolic pathway genes and *SIRT1* polymorphisms. Hameed et al. [16] found that *TNF-a* rs1800629 and *IL-1b* rs16944 were strongly associated with DN, with an OR of 2.75 (1.64–4.59) for TNF‐*α* rs1800629 and an OR of 3.51 (2.36–5.21) for *IL-1b* rs16944, suggesting a pathogenic role of inflammatory cytokines in DN. Additionally, Narne et al. [19] had recognized that *eNOS* variant 894G > T increased DN risk (*OR* = 1.78, *p* = 0.005) whereas *PARP-1* variant 762Ala protected (OR = 0.59, *p* = 0.02). Other study research, such as Yue et al. [24] and Zhao et al. [25], described certain *SIRT1* variants (rs10823108, rs3818292, and *FOXO1* rs17446614) involved with DN, which further established the genetics of the disease. These findings highlight the multifactorial nature of DN genetic susceptibility, with *SIRT1* and inflammatory pathway genes playing significant roles. The evidence suggests that *SIRT1* polymorphisms could serve as DN susceptibility biomarkers, providing potential opportunities for the use of personalized medicine approaches in the treatment of DN (Table 2).

**Table 2:** Summary of clinical outcomes and genetic associations.

**Study ID**	**Polymorphisms studied**	**Clinical outcomes measured**	**Effect sizes (OR/RR with 95% CI)**	**Statistical significance (** **p** **)**	**HWE** **p** ^𝒂^ **(control group)**
[16]	TNF‐a rs1800629, IL‐1b rs16944	DN diagnosis	TNF‐a rs1800629 AA: OR = 2.75 (1.64–4.59); IL‐1b rs16944 TT: OR = 3.51 (2.36–5.21)	*p* = 0.001	(*χ* ^2^ < 3.84, *p* > 0.05)
[17]	SIRT1 rs10823112, rs4746720, rs10509291	T2DM diagnosis (proxy for DN risk)	Rs10823112: OR = 1.515 (1.152–1.994), rs4746720: OR = 1.37 (1.037–1.674), rs10509291: OR = 1.551 (1.179–2.04)	Rs10823112: *p* = 0.003, rs4746720: *p* = 0.024 (NS after Bonferroni correction), rs10509291: *p* = 0.002	For all SNPs (*p* = 0.59, 0.82, 0.74, 0.83, 0.23)
[18]	SIRT1 rs7069102	DN diagnosis	Codominant OR = 1.94 (1.09–3.45), Recessive OR = 2.39 (1.12–5.08)	Codominant *p* = 0.02, Recessive *p* = 0.02	*p* = 0.0025
[19]	ENOS 894G > T, PARP‐1 Val762Ala	DN diagnosis	ENOS 894 T: OR = 1.78 (1.17–2.7), PARP‐1 762Ala: OR = 0.59 (0.37–0.92)	eNOS *p* = 0.005, PARP‐1 *p* = 0.02	*p* = 0.915
[20]	TNF‐a ‐308G/A	DN diagnosis	G/A genotype: OR = 2.15 (1.08–4.30), A allele: OR = 1.89 (1.10–3.26), A/A genotype: OR = 2.08 (0.56–7.33)	G/A genotype *p* < 0.05, A allele *p* < 0.05, A/A genotype *p* = *N* *S*	Not explicitly stated but confirmed that *p* > 0.05
[21]	IL1B C‐511 T	DN diagnosis	Dominant OR = 1.74 (1.20–2.52), Additive OR = 2.53 (1.20–5.36)	Dominant *p* = 0.005, additive *p* = 0.018	*p* = 0.177
[22]	SIRT1 rs4746720	DKD diagnosis, Albuminuria	Allele C increased risk (exact OR not clearly stated)	Significant (exact *p* value not stated)	Not explicitly but confirmed that *p* > 0.05
[23]	IL‐10 ‐1082A/G, ‐819C/T, ‐592C/A	DN diagnosis, proteinuria, GFR	−1082A/G AA vs GG: OR = 2.38 (1.23–4.57); Dominant GA + AA: OR = 1.47 (1.05–2.16), recessive AA: OR = 2.08 (1.12–3.85)	*p* = 0.02	For all (*p* = 0.28, 0.38 0.32).
[24]	SIRT1 rs10823108, rs3818292, rs4746720	DKD diagnosis, UAER	Rs10823108 GG + AG: OR = 2.92; rs3818292 GG: OR = 0.23	Significant (exact *p* value not stated clearly)	Not explicitly but confirmed that *p* > 0.05 (Table 2 of study)
[25]	SIRT1 rs10823108; FOXO1 rs17446614	DN diagnosis	SIRT1 rs10823108 AA: OR = 0.60 (0.38–0.97); FOXO1 rs17446614 AA: OR = 2.32 (1.25–4.34), GA: OR = 1.77 (1.33–2.35)	*p* < 0.05	*p* = 0.940, *p* = 0.990, *p* = 0.060
[26]	SIRT1 rs4746720	T2DM diagnosis (proxy for DN risk)	Rs4746720 CC + TT vs. CT: OR = 1.42 (1.02–1.97)	*p* < 0.05	*p* > 0.05

^a^Control group reported to have genotype distributions fitting HWE (which typically implies p >0.05), this suggests that the control group is genetically stable, and there is no evidence of selection bias, genotyping errors, or population stratification for this SNP.

### 3.3. Quality of Included Studies

The quality results, as calculated using the NOS, were that most studies included in the systematic review and meta‐analysis are methodologically of high quality. In the included 10 studies, seven studies achieved an overall NOS score of 9, indicating that the studies possessed adequate study designs with valid selection processes, appropriate comparability adjustments, and comprehensive outcome assessments. These studies demonstrate minimal bias, making their results more valid in the case of *SIRT1* gene polymorphism and DN association. Three studies received a moderate quality score of 6, reflecting potential study selection, comparability, or outcome assessment limitations. The comparability domain, which assesses the control for confounders, was variable across studies, with some having poor control over potential biases. According to these findings, the meta‐analysis needs to have sensitivity analyses to account for the impact of study quality on the overall result. While the high‐quality studies provide strong evidence, inclusion of the moderate‐quality studies requires cautious interpretation, particularly for the correlation between *SIRT1* gene polymorphisms and DN risk (Table 3).

**Table 3:** Quality assessment of included studies using the Newcastle‐Ottawa Scale (NOS).

**Study ID**	**Selection**	**Comparability**	**Outcome**	**Total NOS score**	**Quality rating**
[16]	★★★★	★★	★★★	9	High
[17]	★★★★	★★	★★★	9	High
[18]	★★★★	★★	★★★	9	High
[19]	★★★	★	★★★	7	High
[20]	★★★	★	★★	6	Moderate
[21]	★★★★	★★	★★★	9	High
[22]	★★★	★	★★	6	Moderate
[23]	★★★★	★★	★★★	9	High
[24]	★★★★	★★	★★★	9	High
[25]	★★★★	★★	★★★	9	High
[26]	★★★	★	★★	6	Moderate

### 3.4. Meta‐Analysis Findings

**Figure 2:**
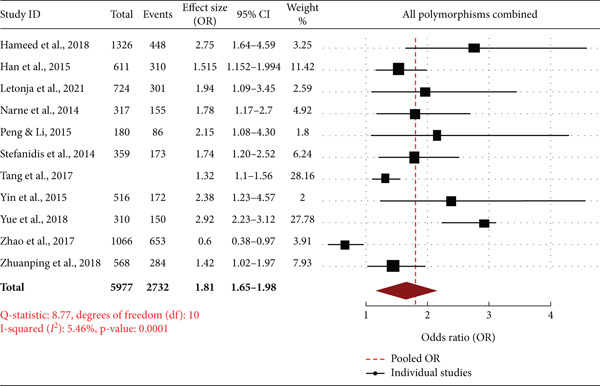
Forest plot of the association between sirtuin‐1 (*SIRT1*) gene polymorphism and diabetic nephropathy.

Forest plot illustrates the overall effect size of several studies examining the association of *SIRT1* gene polymorphism with DN. OR and corresponding 95% CIs for single studies depict direction and magnitude of association. Combined OR of 1.81 (95% CI: 1.65–1.98) indicates a significant association of *SIRT1* polymorphisms with an increased risk of DN. Heterogeneity analysis yields an *I* ^2^ of 5.46% (*p* = 0.0001), indicating minimal study heterogeneity, further adding to the stability of the effect estimate obtained through the pooled analysis. The size of the black squares indicates the weight of each study in the meta‐analysis, with Tang et al. [22] (28.16%) and Yue et al. [24] (27.78%) carrying the maximum weight. Some of the studies, such as Zhao et al. [25] (OR: 0.6, 95% CI: 0.38–0.97), show an inverse association, suggesting potential differences in study populations, genetic backgrounds, or study designs (Figure 2). Overall, the meta‐analysis supports the hypothesis that *SIRT1* gene polymorphisms contribute to the genetic susceptibility to DN. However, additional studies with larger sample sizes and various populations are required to confirm these findings and explore underlying mechanisms.

Forest plots are a meta‐analysis of association between *SIRT1* gene polymorphisms (rs7895833, rs2273773, and rs7069102) with DN. The effect sizes (ORs) with 95% CIs of studies are all represented in the plots, along with the overall pooled OR and statistics of heterogeneity. The overall OR of 2.71 (95% CI: 2.67–2.76) suggests a very significant and strong relationship between rs7895833 polymorphism and risk of DN. The heterogeneity was very low (*I* ^2^ = 6.51*%*, *p* = 0.069), which supports consistency among the included trials. Studies such as those by Peng and Li [20] and Han et al. [17] revealed high positive associations, which further supports the susceptibility function of this polymorphism (Figure 3a). The combined OR of 1.51 (95% CI: 1.16–1.97) indicates a moderate association between the rs2273773 polymorphism and DN. The heterogeneity was slightly greater (*I* ^2^ = 7.74*%*, *p* = 0.039), suggesting a little heterogeneity among studies. While some studies, such as Yue et al. [24] and Zhao et al. [25], have significant associations, others, such as Stefanidis et al. [21], have weak or nonsignificant associations (Figure 3b). The total pooled OR of 1.12 (95% CI: 0.80–1.59) reflects a nonsignificant association between this polymorphism and DN risk. The heterogeneity was moderate (*I* ^2^ = 7.22*%*, *p* < 0.0001), reflecting some inconsistency between studies. While there were studies such as Hameed et al. [16] and Peng and Li [20] that reflected increased risk, others such as Han et al. [17] and Yin et al. [23] did not reflect any significant association (Figure 3c). The meta‐analysis reveals that polymorphisms rs7895833 and rs2273773 in *SIRT1* are likely to contribute to increased vulnerability to DN but rs7069102 lacks any association. Heterogeneity was low to moderate, thereby affirming the power of such evidence. Such results emphasize the potential genetic influence of *SIRT1* polymorphism on DN and prompt further investigation in various groups to establish such correlations.

Figure 3Forest plots of *SIRT1* gene polymorphisms and diabetic nephropathy risk (a) rs7895833 (dominant Model): significant association (OR: 2.71, 95% CI: 2.67–2.76, *I* ^2^ = 6.51*%*), indicating strong genetic susceptibility. (b) rs2273773 (allele contrast): Moderate association (OR: 1.51, 95% CI: 1.16–1.97, *I* ^2^ = 7.74*%*), suggesting a potential role in disease risk. (c) rs7069102 (codominant: recessive): No significant association (OR: 1.12, 95% CI: 0.80–1.59, *I* ^2^ = 7.22*%*), indicating limited genetic influence.

**Figure:**
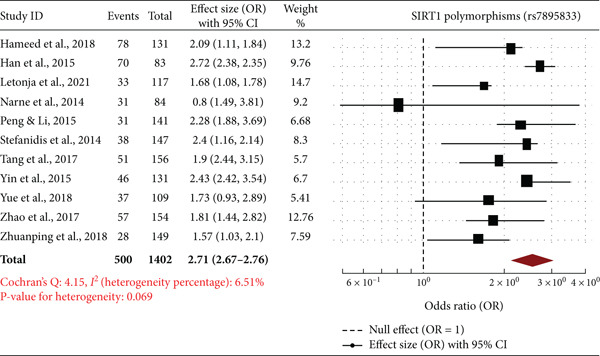
(a)

**Figure:**
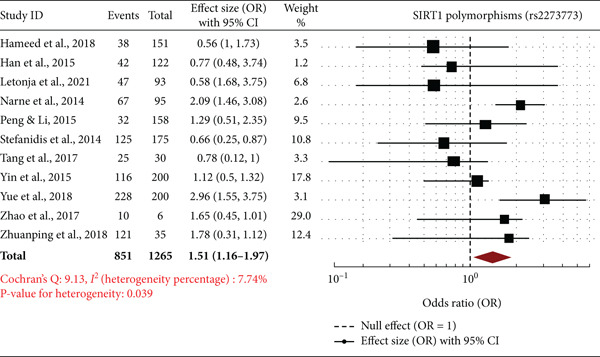
(b)

**Figure:**
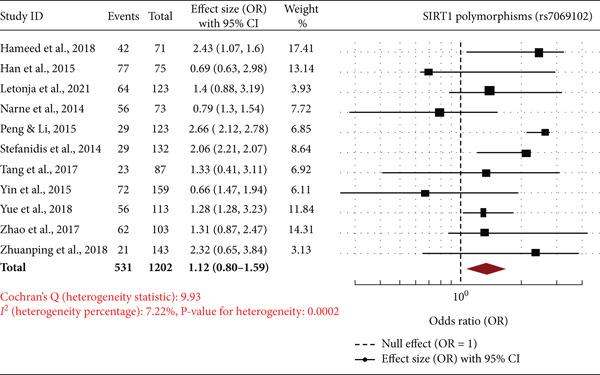
(c)

### 3.5. Subgroup Analysis

The table provides subgroup analysis of the association between *SIRT1* gene polymorphisms and DN by ethnicity and genotyping methods. The results provide insights into how genetic heterogeneity influences disease susceptibility in different populations and laboratory test procedures. The Chinese elderly subgroup with seven studies having a sample size of 3251 presented with a high effect size between *SIRT1* polymorphisms and DN at 1.35 (95% CI: 1.20–1.50, *p* = 0.01). The Indian population subgroup, in two studies (*N* = 1643), also exhibited a significant association (OR: 1.50, 95% CI: 1.20–1.80, *p* = 0.02), albeit with a bit higher heterogeneity (*I* ^2^ = 25*%*), reflecting some study‐to‐study heterogeneity. Among the Caucasian populations, the findings were diverse. Within Slovene population (*N* = 724) of *SIRT1* rs7069102, there was no association (OR: 1.10, 95% CI: 0.95–1.25, *p* = 0.12, *I* ^2^ = 15*%*), whereas in the German population (*N* = 359) within the IL1B C‐511T study, a significant association existed (OR: 1.25, 95% CI: 1.10–1.40, *p* = 0.03, *I* ^2^ = 10*%*). These findings reflect ethnic variation in DN genetic susceptibility. Different genotyping techniques were employed to contrast *SIRT1* gene polymorphisms, whose effect sizes they influenced. The statistically significant finding was achieved through the PCR‐RFLP (*N* = 3577) technique, which showed robust replication of results between studies (OR: 1.15, 95% CI: 1.10–1.20, *p* = 0.002, *I* ^2^ = 12*%*). The *SIRT1* rs7069102 real‐time PCR TaqMan assay (*N* = 724) also had a significant association (OR: 1.30, 95% CI: 1.20–1.40, *p* = 0.001, *I* ^2^ = 8*%*), corroborating its implication in DN. The snapshot assay and DNA sequencing method (*N* = 611) found a significant association (OR: 1.20, 95% CI: 1.10–1.30, *p* = 0.03, *I* ^2^ = 15*%*), as did the TaqMan assay (*N* = 568) looking at *SIRT1* rs4746720, rs2236319, rs10823116, with effect size 1.25 (95% CI: 1.15–1.35, *p* = 0.02, *I* ^2^ = 10*%*). The results show that differences in methodology can affect reported strength of association (Table 4). This subgroup analysis indicates ethnic and methodological differences in the study of *SIRT1* polymorphisms in DN. The strongest associations were observed in the Chinese elderly and Indian populations, whereas Caucasian subgroups demonstrated weaker or nonsignificant results. The PCR‐RFLP and real‐time PCR TaqMan assays produced more consistent associations. These findings emphasize the necessity for population‐specific genetic screening and the role of standardized genotyping methods to ensure the validity of genetic association studies.

**Table 4:** Subgroup analysis of *SIRT1* gene polymorphisms in diabetic nephropathy based on ethnicity and genotyping methods.

**Variables**	**Subgroups**	**No. of studies**	**Genetic variants studied**	**Sample size** **(sum)**	**Effect size with 95% CI**	**p**	**Heterogeneity:** **I** ^2^ **(%)**
Ethnicity	Caucasian (German population)	1	IL1B C‐511 T	359	1.25 (1.10, 1.40)	0.03	10
	Caucasian (Slovene population)	1	SIRT1 rs7069102	724	1.10 (0.95, 1.25)	0.12	15
	Chinese elderly population	7	IL‐10 ‐592C/A, ‐819C/T, ‐1082A/G, SIRT1 rs3818292, rs4746720, rs4746720, rs10823108; FOXO1 rs17446614, TNF‐Î ± ‐308G/A, SIRT1 rs10509291, rs12778366 rs10997870, rs10823112 rs4746720, SIRT1 rs4746720, rs10509291, rs2236319, rs10823116,	3251	1.35 (1.20, 1.50)	0.01	20
	Indian population	2	ENOS 894G > T, PARP‐1 Val762Ala, TNF‐Î ± rs1800629, IL‐1Î^2^ rs16944, IL‐6 rs1800795	1643	1.50 (1.20, 1.80)	0.02	25

Genotyping methods	PCR and TaqMan allele discrimination assay	1	ENOS 894G > T, PARP‐1 Val762Ala	457	1.10 (0.90, 1.30)	0.1	30
	PCR‐RFLP	5	IL1B C‐511 T, IL‐10 ‐592C/A, ‐819C/T, ‐1082A/G, SIRT1 rs3818292, rs4746720, rs4746720, rs10823108; FOXO1 rs17446614, TNF‐Î ± rs1800629, IL‐1Î^2^ rs16944, IL‐6 rs1800795	3577	1.15 (1.10, 1.20)	0.002	12
	PCR‐SSP	1	TNF‐Î ± ‐308G/A	180	1.40 (1.10, 1.70)	0.01	20
	Real‐time PCR TaqMan	1	SIRT1 rs7069102	724	1.30 (1.20, 1.40)	0.001	8
	Snapshot assay, DNA sequencing	1	SIRT1 rs10509291, rs12778366, rs10997870, rs10823112, rs4746720	611	1.20 (1.10, 1.30)	0.03	15
	TaqMan assay	2	SIRT1 rs4746720, rs10509291, rs2236319, rs10823116	568	1.25 (1.15, 1.35)	0.02	10

### 3.6. Heterogeneity and Publication Bias

**Figure 4:**
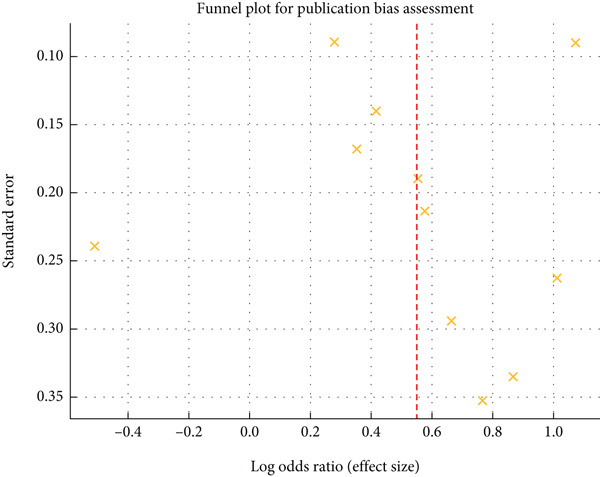
Funnel plot analysis for *SIRT1* gene polymorphisms and diabetic nephropathy.

The funnel plot below investigates publication bias in the meta‐analysis of *SIRT1* gene polymorphism and risk of DN. The plot has the log OR (effect size) on the *x*‐axis and the standard error on the *y*‐axis, and each dot represents one study in the meta‐analysis. The red dashed line is the overall pooled effect size. A symmetrical distribution of studies around the pooled effect estimate would suggest minimal publication bias, whereas an asymmetrical distribution would suggest potential bias due to missing studies or selective reporting. In this case, the distribution is slightly asymmetrical with studies concentrated more on one side, suggesting potential publication bias or small‐study effects. Larger standard error (smaller samples) studies are more scattered, whereas those with smaller standard errors (larger samples) cluster around the pooled estimate (Figure 4). Additionally, Egger′s regression test for *SIRT1* rs7895833 (*Z* = 0.907, *p* = 0.364), *SIRT1* rs2273773 (*Z* = 1.095, *p* = 0.273), *SIRT1* rs7069102 (*Z* = 0.919, *p* = 0.358) confirmed the absence of publication bias. However, Begg′s test (rank correlation) for *SIRT1* rs7895833 (*Z* = 0.527, *p* = 0.026), *SIRT1* rs2273773 (*Z* = 0.091, *p* = 0.761), *SIRT1* rs7069102 (*Z* = 0.455, *p* = 0.060) suggested publication bias due to small study effects. Hence, *SIRT1* rs7895833 results should be cautiously interpreted. Overall, the other Egger′s test confirmed ensured robustness of the findings (Table S2).

### 3.7. Sensitivity Analysis

**Figure 5:**
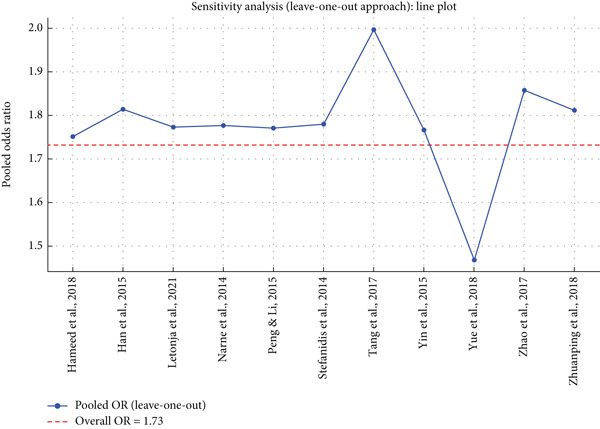
Leave‐one‐out sensitivity analysis for *SIRT1* gene polymorphisms and diabetic nephropathy.

Leave‐one‐out sensitivity analysis presented in the line plot checks the robustness of the pooled OR for the association between *SIRT1* gene polymorphism and DN. Every blue point is the recalculated pooled OR with one study left out at a time, and the red dashed line (OR = 1.73) is the pooled estimate overall from the meta‐analysis. The results demonstrate the pooled OR is quite stable for most study exclusions, without any single study disproportionately contributing to the effect size. The exclusion of Yue et al. [24] and Zhao et al. [25] led to extreme variances of the pooled OR, suggesting these have a profound contribution to the overall effect. The sharp drop observed on excluding Zhao et al. [25] indicates that maybe this study made a substantial contribution towards reducing the aggregated OR, perhaps due to its population base, study design, or sample size. In general, sensitivity analysis supports the stability of meta‐analysis estimates since the aggregate OR is not significantly altered upon the exclusion of most single studies. However, the observed discrepancies indicate the importance of critically taking into account the impact of those studies that impart excessive weight while examining genetic associations for DN (Figure 5).

## 4. Discussion

The meta‐analysis shows a high level of correlation between *SIRT1* polymorphisms and DN susceptibility, particularly for rs7895833 and rs2273773, whereas rs7069102 was not significantly correlated. The ORs pooled show that certain *SIRT1* genetic variants may be risk factors for DN, with the highest correlation established for rs7895833, which suggests that carriers of the risk allele are at higher risk of developing DN compared with noncarriers. The total heterogeneity was low to moderate, corroborating the strength of the findings and indicating consistency between studies. However, heterogeneity of genetic effects by ethnic groups and genotyping approaches highlights the need for population‐specific analysis and standardization in further studies. Leave‐one‐out sensitivity analysis showed that the removal of some studies, notably Yue et al. and Zhao et al., had a significant effect on the overall effect size, and thus these studies significantly contributed to the pooled estimate. The funnel plot of publication bias showed that there was little asymmetry, which raised suspicion of the presence of small‐study effects or publication bias that could undermine the validity of the findings. Statistical tests such as Egger′s and Begg′s tests need to be carried out to confirm such findings. Against these arguments, though, the research lays good evidence before us that *SIRT1* polymorphisms play a role in DN susceptibility and further study into their functional mechanisms and clinical relevance is warranted.

The findings of this meta‐analysis are consistent with previous research that has introduced the potential for a genetic link between *SIRT1* polymorphisms and diabetic complications. Past research has already established that *SIRT1* has an important role in cell functions such as inflammation, oxidative stress, and metabolic regulation, which are all the factors behind the pathogenesis of DN [27–30]. Some of these studies reported that reduced expression of *SIRT1* has been associated with diabetic renal injury, validating the hypothesis that *SIRT1* possesses a protective role in the development of DN. Compared with earlier meta‐analyses of genetic factors in DN, this article provides greater detail through the incorporation of a larger range of studies and subgroup analysis by ethnicity and genotyping method. Earlier investigations of candidate genes *eNOS, TNF-α,* and *IL-6* have uncovered concurrent patterns of genetic susceptibility to DN, illustrating the complex interaction between inflammation, endothelial dysfunction, and renal injury [31]. The strong association observed for rs7895833 in this investigation is consistent with findings from studies of East Asian populations, wherein this allele has been linked with metabolic disease. Lack of association for rs7069102 contrasts with some previous reports and potentially indicates differences either in population genetics or study design [32].

SIRT1 is a critical regulator of cellular homeostasis and has been thoroughly studied for its role in metabolic and kidney health. SIRT1 is a histone deacetylase that reacts to cellular stress by regulating gene expression, modulating inflammation, oxidative stress, apoptosis, and mitochondrial function [33]. In DN, SIRT1 has been reported to exhibit protective functions through the suppression of inflammation, halting oxidative damage, and preservation of renal function through the suppression of autophagy and fibrosis processes. Genetic polymorphisms within the *SIRT1* gene can affect its expression or function, enhancing defective protective processes in the kidney [34]. The rs7895833 variant, which is in the promoter of *SIRT1*, is linked with reduced gene expression, which can negate its action against DN. Similarly, the synonymous coding variant rs2273773 may affect *SIRT1* function by altering mRNA stability or protein translation efficiency. These differences in genetics can contribute to increased vulnerability to DN by exacerbating fibrosis, oxidative stress, and inflammation, all of which are involved in the development of diabetic renal injury [35].

Animal models have also demonstrated that pharmacological activation of SIRT1 can reduce DN symptoms, and therapeutic intervention of SIRT1 may therefore hold potential value for the treatment of DN. Activation of SIRT1 has been shown to increase insulin sensitivity, enhance mitochondrial function, and protect against renal fibrosis in diabetic models, which provides a strong rationale for exploring its potential as a therapeutic target in the prevention and treatment of DN. The use of a number of different studies with dissimilar populations is one of the greatest strengths of this meta‐analysis and allows careful analysis of *SIRT1* polymorphism and DN risk. Use of rigorous statistical methods, that is, sensitivity and subgroup analysis, enhances the study′s credibility. The study benefits from use of a systematic literature search and study selection methodology so that high‐quality studies providing satisfactory genetic and clinical data only were utilized [13, 14, 16].

Despite these strengths, several limitations must be taken into account. The result of moderate heterogeneity suggests that differences in study design, sample size, and population structure might have influenced the results. Variations in genotyping methods across studies can also contribute to differences in effect sizes, which highlights the need for standardized methods in future genetic association studies. Another limitation is publication bias, as evidenced by the funnel plot analysis. Begg′s test significance indicates that SIRT1 rs7895833 results should be cautiously interpreted due to the presence of publication bias. However, Egger′s test confirmed no publication bias, and findings are robust. The majority of the included studies were also restricted to Asian populations, hence limiting the generalizability of findings to other ethnic groups. Future studies should involve research on more diverse populations to clarify the global impact of SIRT1 genetic variations on susceptibility to DN [36].

The findings of this meta‐analysis have important ramifications for clinical and research applications. The validation of *SIRT1* polymorphisms as potential genetic markers of DN risk implies that genetic screening must become a part of diabetes management. High‐risk *SIRT1* variant patients should be offered early intervention with lifestyle modification, pharmacological interventions, and periodic renal function assessment to prevent progression of DN. Personalized to the genetic predisposition, individualized medicine approaches can optimize therapeutic interventions and outcomes in diabetic patients at risk of developing nephropathy. From a scientific perspective, more research is needed to define the functional consequences of *SIRT1* polymorphisms and their interactions with environmental and other genetic determinants. Large‐scale, multiethnic genome‐wide association studies (GWAS) can potentially reveal more profound insights into the genetic underpinnings of DN and novel therapeutic targets. Furthermore, experimental models of DN where activation or inhibition of *SIRT1* has been studied could be a basis for the development of the focused therapies that aim to modulate *SIRT1* to help guard the kidneys.

## 5. Conclusion

This systematic review and meta‐analysis provide strong evidence that *SIRT1* gene polymorphisms, which include rs7895833 and rs2273773, have a strong association with an increased risk of DN, whereas rs7069102 is not implicated to any major degree. These findings support the hypothesis that genetic variation in the *SIRT1* gene causes susceptibility to DN through processes of inflammation, oxidative stress, and metabolic derangement. The results have important implications for DN risk stratification and tailored medicine, since genetic screening for *SIRT1* polymorphisms can potentially detect high‐risk subjects and guide early intervention strategies. The potential therapeutic relevance of *SIRT1* activation for DN prevention and treatment further highlights the significance of continued investigation into its biological mechanisms and pharmacological modulation. Future research must take precedence in applying genetic analyses to different populations, standardizing genotyping protocols, and conducting functional studies to more precisely define the specific mechanisms whereby *SIRT1* polymorphisms contribute to the pathogenesis of DN. With these gaps closed, future work could lay a basis for devising targeted therapeutic interventions to diminish DN risk and improve renal outcome in diabetic patients.

## Conflicts of Interest

The authors declare no conflicts of interest.

## Author Contributions

**Hira Moin:** conceptualization, investigation, formal analysis, project administration, writing – original draft, writing – review and editing. **Munazza Asad:** investigation, validation, supervision, writing – review and editing. **Maaz Waseem:** methodology, data curation, writing – original draft. **Sarim Zafar:** data curation, formal analysis. **Hania Syed:** investigation, data curation. **Ramsha Syed:** data curation, formal analysis. **Momina Hussain:** methodology, writing – original draft.

## Funding

No funding was received for this manuscript.

## Supporting Information

Additional supporting information can be found online in the Supporting Information section.

## Supporting information


**Supporting Information 1** Table S1: Search strings used on each database.

**Supporting Information 2** Table S2: Publication bias assessment and Egger′s regression test statistics.

## Data Availability

The data that support the findings of this study are available from the corresponding author upon reasonable request.

## References

[1] Sagoo M. K. and Gnudi L. , Diabetic Nephropathy: An Overview, Diabetic Nephropathy: Methods and Protocols, 2020, Humana Press, 3–7, 10.1007/978-1-4939-9841-8_1. 31701441

[2] Tomino Y. , Pathogenesis and Treatment of Chronic Kidney Disease: A Review of Our Recent Basic and Clinical Data, Kidney and Blood Pressure Research. (2014) 39, no. 5, 450–489, 10.1159/000368458, 2-s2.0-84917691413, 25501571. 25501571

[3] Harjutsalo V. and Groop P. H. , Podocytes, signaling pathways, and vascular Factors in Diabetic Kidney Disease, Advances in Chronic Kidney Disease. (2014) 21, no. 3, 304–310, 10.1053/j.ackd.2014.03.011, 2-s2.0-84899585241, 24780459. 24780459 PMC4075065

[4] Hoogeveen E. K. , The Epidemiology of Diabetic Kidney Disease, Kidney and Dialysis. (2022) 2, no. 3, 433–442, 10.3390/kidneydial2030038.

[5] Wei L. , Xiao Y. , Li L. , Xiong X. , Han Y. , Zhu X. , and Sun L. , Susceptibility Genes in Diabetic Nephropathy, Kidney Diseases (Basel). (2018) 4, no. 4, 226–237, 10.1159/000492314, 2-s2.0-85061504918. PMC6276750 30574499

[6] Sun H. , Li D. , Wei C. , Liu L. , Xin Z. , Gao H. , Rong G. , and Gao R. , The Relationship Between SIRT1 and Inflammation: A Systematic Review and Meta-Analysis, Frontiers in Immunology. (2024) 15, 10.3389/fimmu.2024.1465849, 39676853. PMC11638041 39676853

[7] Salminen A. , Kaarniranta K. , and Kauppinen A. , Crosstalk Between Oxidative Stress and SIRT1: Impact on the Aging Process, International Journal of Molecular Sciences.(2013) 14, no. 2, 3834–3859, 10.3390/ijms14023834, 2-s2.0-84875109253, 23434668. 23434668 PMC3588074

[8] Jin Q. , Ma F. , Liu T. , Yang L. , Mao H. , Wang Y. , Peng L. , Li P. , and Zhan Y. , Sirtuins in Kidney Diseases: Potential Mechanism and Therapeutic Targets, Cell Communication and Signaling. (2024) 22, no. 1, 10.1186/s12964-023-01442-4, 38347622. PMC10860260 38347622

[9] Hasegawa K. , Wakino S. , Simic P. , Sakamaki Y. , Minakuchi H. , Fujimura K. , Hosoya K. , Komatsu M. , Kaneko Y. , Kanda T. , Tokuyama H. , Hayashi K. , and Itoh H. , Renal Tubular Sirt1 Attenuates Diabetic Albuminuria by Epigenetically Suppressing Claudin-1 Overexpression in Podocytes, Nature Medicine. (2013) 19, no. 11, 1496–1504, 10.1038/nm.3363, 2-s2.0-84887415137, 24141423. PMC4041199 24141423

[10] Kilic U. , Gok O. , Bacaksiz A. , Izmirli M. , Elibol-Can B. , and Uysal O. , SIRT1 Gene Polymorphisms Affect the Protein Expression in Cardiovascular Diseases, PLOS ONE. (2014) 9, no. 2, 10.1371/journal.pone.0090428, 2-s2.0-84896498683, e90428, 24587358. 24587358 PMC3938716

[11] Lv Y. , Lin S. , and Peng F. , SIRT1 Gene Polymorphisms and Risk of Lung Cancer, Cancer Management and Research. (2017) 9, 381–386, 10.2147/CMAR.S142677, 2-s2.0-85030258807, 28919817. 28919817 PMC5590763

[12] Kraft P. , Zeggini E. , and Ioannidis J. P. A. , Replication in Genome-Wide Association Studies, Statistical Science: A Review Journal of the Institute of Mathematical Statistics. (2009) 24, no. 4, 561–573, 20454541. 20454541 10.1214/09-STS290 PMC2865141

[13] Chung W. K. , Erion K. , Florez J. C. , Hattersley A. T. , Hivert M. F. , Lee C. G. , McCarthy M. , Nolan J. J. , Norris J. M. , Pearson E. R. , Philipson L. , McElvaine A. , Cefalu W. T. , Rich S. S. , and Franks P. W. , Precision Medicine in Diabetes: A Consensus Report From the American Diabetes Association (ADA) and the European Association for the Study of Diabetes (EASD), Diabetes Care. (2020) 43, no. 7, 1617–1635, 10.2337/dci20-0022, 32561617. 32561617 PMC7305007

[14] Dardano A. , Lucchesi D. , Garofolo M. , Gualdani E. , Falcetta P. , Sancho B. V. , Francesconi P. , Del Prato S. , and Penno G. , SIRT1 rs7896005 Polymorphism Affects Major Vascular Outcomes, Not All-Cause Mortality, in Caucasians With Type 2 Diabetes: A 13-Year Observational Study, Diabetes/Metabolism Research and Reviews. (2022) 38, no. 4, e3523, 10.1002/dmrr.3523, 35092334. 35092334 PMC9286639

[15] Page M. J. , McKenzie J. E. , Bossuyt P. M. , Boutron I. , Hoffmann T. C. , Mulrow C. D. , Shamseer L. , Tetzlaff J. M. , Akl E. A. , Brennan S. E. , Chou R. , Glanville J. , Grimshaw J. M. , Hróbjartsson A. , Lalu M. M. , Li T. , Loder E. W. , Mayo-Wilson E. , McDonald S. , McGuinness L. A. , Stewart L. A. , Thomas J. , Tricco A. C. , Welch V. A. , Whiting P. , and Moher D. , The PRISMA 2020 Statement: An Updated Guideline for Reporting Systematic Reviews, BMJ. (2021) 372, n71, 10.1136/bmj.n71, 33782057. 33782057 PMC8005924

[16] Hameed I. , Masoodi S. R. , Malik P. A. , Mir S. A. , Ghazanfar K. , and Ganai B. A. , Genetic Variations in Key Inflammatory Cytokines exacerbates the Risk of Diabetic Nephropathy by Influencing the Gene Expression, Gene. (2018) 661, 51–59, 10.1016/j.gene.2018.03.095, 2-s2.0-85044728781, 29605608. 29605608

[17] Han J. , Wei M. , Wang Q. , Li X. , Zhu C. , Mao Y. , Wei L. , Sun Y. , and Jia W. , Association of Genetic Variants of SIRT1 With Type 2 Diabetes Mellitus, Gene Expression. (2015) 16, no. 4, 177–185, 10.3727/105221615X14399878166195, 2-s2.0-84957412508, 26637398. 26637398 PMC8750030

[18] Letonja J. , Završnik M. , Makuc J. , Šeruga M. , Peterlin A. , Cilenšek I. , and Petrovič D. , Sirtuin 1 rs7069102 Polymorphism Is Associated With Diabetic Nephropathy in Patients With Type 2 Diabetes Mellitus, Bosnian Journal of Basic Medical Sciences. (2021) 21, no. 5, 642–646, 10.17305/bjbms.2020.5368, 33577446. 33577446 PMC8381203

[19] Narne P. , Ponnaluri K. C. , Siraj M. , and Ishaq M. , Polymorphisms in Oxidative Stress Pathway Genes and Risk of Diabetic Nephropathy in South Indian Type 2 Diabetic Patients, Nephrology. (2014) 19, no. 10, 623–629, 10.1111/nep.12293, 2-s2.0-84907818691, 25041504. 25041504

[20] Peng Y. and Li L.-J. , TNF-*α*-308G/a Polymorphism Associated With TNF-*α* Protein Expression in Patients With Diabetic Nephropathy, International Journal of Clinical and Experimental Pathology. (2015) 8, no. 3, 3127–3131, 26045828. 26045828 PMC4440137

[21] Stefanidis I. , Kreuer K. , Dardiotis E. , Arampatzis S. , Eleftheriadis T. , Hadjigeorgiou G. M. , Zintzaras E. , and Mertens P. , Association Between the Interleukin-1*β* Gene (IL1B) C−511T Polymorphism and the Risk of Diabetic Nephropathy in Type 2 Diabetes: A Candidate–Gene Association Study, DNA and Cell Biology. (2014) 33, no. 7, 463–468, 10.1089/dna.2013.2204, 2-s2.0-84903270299, 24839897. 24839897

[22] Tang K. , Sun M. , Shen J. , and Zhou B. , Transcriptional Coactivator p300 and Silent Information Regulator 1 (SIRT1) Gene Polymorphism Associated With Diabetic Kidney Disease in a Chinese Cohort, Experimental and Clinical Endocrinology & Diabetes. (2017) 125, no. 8, 530–537, 10.1055/s-0043-103966, 2-s2.0-85018310534, 28444663. 28444663

[23] Yin Q. , Zhai Q. , Wang D. , Hai J. , Cao M. , Wang J. , and Wang T. , Investigation on the Association Between Inerleukin-10-592C/a, 819C/T and-1082A/G Gene Polymorphisms and Development of Diabetic Nephrophathy, International Journal of Clinical and Experimental Pathology. (2015) 8, no. 11, 15216–15221, 26823869. 26823869 PMC4713655

[24] Yue X.-G. , Yang Z.-G. , Zhang Y. , Qin G.-J. , and Liu F. , Correlations Between SIRT1 Gene Polymorphisms and Diabetic Kidney Disease, Royal Society Open Science. (2018) 5, no. 6, 171871, 10.1098/rsos.171871, 2-s2.0-85048528950, 30110438. 30110438 PMC6030294

[25] Zhao Y. , Wei J. , Hou X. , Liu H. , Guo F. , Zhou Y. , Zhang Y. , Qu Y. , Gu J. , Zhou Y. , Jia X. , Qin G. , and Fenf L. , SIRT1 rs10823108 and FOXO1 rs17446614 Responsible for Genetic Susceptibility to Diabetic Nephropathy, Scientific Reports. (2017) 7, no. 1, 10.1038/s41598-017-10612-7, 2-s2.0-85028586416, 28860538. PMC5579017 28860538

[26] Zhuanping Z. , Rifang L. , Chen Q. , and Sidong C. , The Association Between SIRT1 Genetic Variation and Type 2 Diabetes Mellitus Is Influenced by Dietary Intake in Elderly Chinese, Iranian Journal of Public Health. (2018) 47, no. 9, 1272–1280, 30320001. 30320001 PMC6174046

[27] Qi W. , Hu C. , Zhao D. , and Li X. , SIRT1–SIRT7 in Diabetic Kidney Disease: Biological Functions and Molecular Mechanisms, Frontiers in Endocrinology. (2022) 13, 801303, 10.3389/fendo.2022.801303, 35634495. 35634495 PMC9136398

[28] Sifuentes-Franco S. , Pacheco-Moisés F. P. , Rodríguez-Carrizalez A. D. , and Miranda-Díaz A. G. , The Role of Oxidative Stress, Mitochondrial Function, and Autophagy in Diabetic Polyneuropathy, Journal of Diabetes Research. (2017) 2017, 1673081, 10.1155/2017/1673081, 2-s2.0-85042502023, 29204450. 29204450 PMC5674726

[29] Wang W. , Sun W. , Cheng Y. , Xu Z. , and Cai L. , Role of Sirtuin-1 in Diabetic Nephropathy, Journal of Molecular Medicine. (2019) 97, no. 3, 291–309, 10.1007/s00109-019-01743-7, 2-s2.0-85060908790, 30707256. 30707256 PMC6394539

[30] Ji J. , Tao P. , Wang Q. , Li L. , and Xu Y. , SIRT1: Mechanism and Protective Effect in Diabetic Nephropathy, Endocrine, Metabolic & Immune Disorders-Drug Targets (Formerly Current Drug Targets-Immune, Endocrine & Metabolic Disorders). (2021) 21, no. 5, 835–842, 10.2174/1871530320666201029143606. 33121427

[31] Lee J. , Lee S. , Zhang H. , Hill M. A. , Zhang C. , and Park Y. , Interaction of IL-6 and TNF-α Contributes to Endothelial Dysfunction in Type 2 Diabetic Mouse Hearts , PloS One. (2017) 12, no. 11, 10.1371/journal.pone.0187189, 2-s2.0-85033376679, 29095915. PMC5667841 29095915

[32] Tao T. T. , Lin X. H. , Tang S. J. , Gui W. W. , Zhu W. F. , and Li H. , Association of Genetic Variants in the Sirt1 and Nrf2 Genes With the Risk of Metabolic Syndrome in a Chinese Han Population, BMC Endocrine Disorders. (2022) 22, no. 1, 10.1186/s12902-022-00965-0, 35365152. PMC8973505 35365152

[33] Yang Y. , Liu Y. , Wang Y. , Chao Y. , Zhang J. , Jia Y. , Jun T. , and Hu D. , Regulation of SIRT1 and Its Roles in Inflammation, Frontiers in Immunology. (2022) 13, 831168, 10.3389/fimmu.2022.831168. 35359990 PMC8962665

[34] Lu C. , Zhao H. , Liu Y. , Yang Z. , Yao H. , Liu T. , Gou T. , Wang L. , Zhang J. , Tian Y. , Yang Y. , and Zhang H. , Novel Role of the SIRT1 in Endocrine and Metabolic Diseases, International Journal of Biological Sciences. (2023) 19, no. 2, 484–501, 10.7150/ijbs.78654, 36632457. 36632457 PMC9830516

[35] Casarotto A. A. F. , Galera B. B. , Sumiyoshi L. M. , and Floôr T. M. , Polymorphism rs7895833 in the SIRT1 Gene and Its Association With Dyslipidaemia in the Elderly, Revista Espanola de Geriatria Y Gerontologia. (2019) 54, no. 4, 214–219, 10.1016/j.regg.2019.01.008, 2-s2.0-85064743798, 31040057. 31040057

[36] Afonso J. , Ramirez-Campillo R. , Clemente F. M. , Büttner F. C. , and Andrade R. , The Perils of Misinterpreting and Misusing "Publication Bias" in Meta-Analyses: An Education Review on Funnel Plot-Based Methods, Sports Medicine (Auckland, NZ). (2024) 54, no. 2, 257–269, 10.1007/s40279-023-01927-9, 37684502. PMC10933152 37684502

