# Supplementary material for: Deciphering the Role of Sirtuin‐1 Gene Polymorphism in Diabetic Nephropathy: A Systematic Review and Meta‐Analysis
Source: J Diabetes Res. 2026 Jan 29;2026:5528647. doi: 10.1155/jdr/5528647 (PMC12853138; doi:10.1155/jdr/5528647)
Supplement: Supplementary file 1 — Supporting Information 1 Table S1: Search strings used on each database. [file JDR-2026-5528647-s001.docx]

**SUPPLEMENTARY FILE 1**

**Table S1 : Search strategy used on respective database**

| **Source** | **String** | **Hits** |
| --- | --- | --- |
| **Pubmed** | (("Diabetic Nephropathy"[Mesh] OR "Diabetic Kidney Disease" OR "Diabetic Nephropathy" OR "DN" OR "Diabetes-related renal disease" OR "Diabetes nephropathy") AND ("Genetic Polymorphism"[Mesh] OR "SNP" OR "Single Nucleotide Polymorphism" OR "Genetic Association Study" OR "Genome-Wide Association Study" OR "Genomic Variation" OR "Genotype Frequencies" OR "Haplotypes")) | 1184 |
| **Scopus** | (TITLE-ABS-KEY("Diabetic Nephropathy" OR "Diabetic Kidney Disease" OR "DN" OR "Diabetes-related renal disease")  AND TITLE-ABS-KEY("SIRT1" OR "Sirtuin-1" OR "SIRT1 gene" OR "SIRT1 polymorphism" OR "SIRT1 variant" OR "rs7895833" OR "rs2273773" OR "rs7069102" OR "SIRT1 expression" OR "SIRT1 mutation")  AND TITLE-ABS-KEY("Genetic Polymorphism" OR "SNP" OR "Single Nucleotide Polymorphism" OR "Genome-Wide Association Study" OR "Genetic Risk" OR "Genotype Frequencies" OR "Haplotypes" OR "Genetic Susceptibility")) | 1310 |
| **Cochrane Library** | ("Diabetic Nephropathy" OR "Diabetic Kidney Disease" OR "Diabetes-related renal disease") AND ("Genetic Association Study" OR "SNP" OR "Genome-Wide Association Study" OR "Genetic Susceptibility" OR "Genotype Frequencies") | 17 |
| **Embase** | ('diabetic nephropathy'/exp OR 'diabetic kidney disease' OR 'DN' OR 'diabetes-related renal disease')  AND ('SIRT1'/exp OR 'Sirtuin 1' OR 'SIRT1 gene' OR 'SIRT1 polymorphism' OR 'SIRT1 variant' OR 'rs7895833' OR 'rs2273773' OR 'rs7069102' OR 'SIRT1 mutation')  AND ('genetic polymorphism'/exp OR 'SNP' OR 'single nucleotide polymorphism' OR 'genome-wide association study' OR 'genetic susceptibility' OR 'haplotypes') | 27 |
| **Total Hits** |  | 2538 |
