# Supplementary material for: Deciphering the Role of Sirtuin‐1 Gene Polymorphism in Diabetic Nephropathy: A Systematic Review and Meta‐Analysis
Source: J Diabetes Res. 2026 Jan 29;2026:5528647. doi: 10.1155/jdr/5528647 (PMC12853138; doi:10.1155/jdr/5528647)
Supplement: Supplementary file 2 — Supporting Information 2 Table S2: Publication bias assessment and Egger′s regression test statistics. [file JDR-2026-5528647-s002.docx]

**SUPPLEMENTARY FILE 2**

**Table S2: Publication bias assessment**

| **Publication bias assessment (SRIT1 7895833)** | | | | | | | | | | | | | |
| --- | --- | --- | --- | --- | --- | --- | --- | --- | --- | --- | --- | --- | --- |
| **Fail-Safe N Analysis (File Drawer Analysis)** | | | | | | | | | | | | | |
| **Fail-safe N** | | | | | | | | | **p** | | | | |
| **3855.000** | |  | | | | | | | **< .001** | | | |  |
| **Note. Fail-safe N Calculation Using the Rosenthal Approach** | | | | | | | | | | | | | |
| **Rank Correlation Test for Funnel Plot Asymmetry** | | | | | | | | | | | | | |
| **Kendall's Tau** | | | | | | | **p** | | | | | | |
| **0.527** | | |  | | | | | | | **0.026** | | | |
| **Regression Test for Funnel Plot Asymmetry** | | | | | | | | | | | | | |
| **Z** | | | | | **p** | | | | | | | | |
| **0.907** |  | | | | **0.364** | | | | | |  | | |
| **Publication bias assessment (SRIT1 rs2273773)** | | | | | | | | | | | | | |
| Fail-Safe N Analysis (File Drawer Analysis) | | | | | | | | | | | | | |
| **Fail-safe N** | | | | | | | | | | | | **p** | |
| 5983.000 | | | | | | | | | | | | < .001 | |
| Note. Fail-safe N Calculation Using the Rosenthal Approach | | | | | | | | | | | | | |
| Rank Correlation (Begg’s) Test for Funnel Plot Asymmetry | | | | | | | | | | | | | |
| **Kendall's Tau** | | | | | | | | | | | | **p** | |
| 0.091 | | | | | | | | | | | | 0.761 | |
| Regression Test for Funnel Plot Asymmetry | | | | | | | | | | | | | |
| **Z** | | | | | | | | | | | | **p** | |
| 1.095 | | | | | | | | | | | | 0.273 | |
| **Publication Bias Assessment (SRIT1 rs7069102)** | | | | | | | | | | | | | |
| Fail-Safe N Analysis (File Drawer Analysis) | | | | | | | | | | | | | |
| **Fail-safe N** | | | | | | | | **p** | | | | | |
| 5590.000 | | | | | | | | < .001 | | | | | |
| Note. Fail-safe N Calculation Using the Rosenthal Approach | | | | | | | | | | | | | |
| Rank Correlation Test for Funnel Plot Asymmetry | | | | | | | | | | | | | |
| **Kendall's Tau** | | | | | | **p** | | | | | | | |
| 0.455 | | | | | | 0.060 | | | | | | | |
| Regression Test for Funnel Plot Asymmetry | | | | | | | | | | | | | |
| **Z** | | | | **p** | | | | | | | | | |
| 0.919 | | | | 0.358 | | | | | | | | | |
